# The Future of Smallpox Vaccination: is MVA the key?

**DOI:** 10.1186/1476-9433-4-2

**Published:** 2005-03-01

**Authors:** Mark K Slifka

**Affiliations:** 1 Vaccine and Gene Therapy Institute, Oregon Health & Sciences University, 505 NW 185^th ^Avenue, Beaverton, OR 97006, USA

## Abstract

Eradication of the smallpox virus through extensive global vaccination efforts has resulted in one of the most important breakthroughs in medical history, saving countless lives from the severe morbidity and mortality that is associated with this disease. Although smallpox is now extinct in nature, laboratory stocks of this virus still remain and the subject of smallpox vaccination has gained renewed attention due to the potential risk that smallpox may be used as a biological weapon by terrorists or rogue states. Despite having the longest history of any modern vaccine, there is still much to be learned about smallpox vaccination and the correlates of protection remain to be formally defined. This Commentary will discuss the strengths and weaknesses of traditional smallpox vaccination in comparison with immunization using modified vaccinia virus Ankura (MVA), a non-replicating virus with a strong safety record but weakened immunogenicity.

## Introduction

Smallpox (Variola major) is a virus that no longer exists in the wild, but during its reign it caused 20–30% mortality in previously unvaccinated individuals and often left survivors with deeply pitted scars for life [1]. The last case of smallpox in the U.S. occurred in 1949 and the last case of naturally occurring smallpox in the world occurred in Somalia in 1977. Smallpox has no known animal reservoir, so in the absence of any more natural cases of human smallpox being recorded after 1977, the virus was considered fully eradicated in 1980 [1]. Despite extinction in nature, smallpox virus stocks still reside in secure locations within the U.S. and Russia but it is impossible to know if other undeclared stocks of smallpox remain in other countries [2]. Moreover, in the age of genetic engineering it is possible that more virulent strains of smallpox or other potentially dangerous orthopoxviruses could be developed and unleashed in an effort of bioterrorism. Although the potential for developing a pathogen more lethal than wild smallpox is theoretically possible [2,3] it would by no means be a simple task to undertake and the outcome would likewise be uncertain [4,5]. Nevertheless, smallpox is considered a potential risk to national security and efforts are underway to prepare the United States and several other countries for a deliberate release of smallpox as a biological weapon.

The first line of defense against smallpox is vaccination. Smallpox vaccination is highly effective at protecting against lethal infection and even if only partial protective immunity is attained, this often still results in survival and decreased viral spread to others (Chapter 4, pages 189–90 of [1]). The smallpox vaccine was discovered by Edward Jenner, who was the first to prove that infection by cowpox resulted in protective cross-reactive immunity against smallpox [6]. Dr. Jenner not only demonstrated that cowpox could induce protective immunity, but in answer to critics of his day who argued that this form of immunity would only be short-lived, he demonstrated full protection against smallpox in several individuals at 25, 27, 31, 38, and even 53 years after cowpox infection [6,7]. It is not a coincidence that such long-term time points were examined. As Dr. Jenner noted, *"I have purposely selected several cases in which the disease *[i.e. cowpox] *had appeared at a very distant period previous to the experiments made with variolous matter, to show that the change produced in the constitution is not affected by time." *[6].

Following the elegant studies initiated by Edward Jenner, the world was eventually freed of the scourge of smallpox following a massive global eradication campaign [1] and although hugely successful, there were still many questions that were left unanswered. These questions represent the topics of this commentary. For instance, by then end of the 1960's it was realized that smallpox vaccination was the cause of a substantial number of adverse events and resulted in a lethal infection in approximately one out of one million people who received this live viral vaccine. In Germany, an extremely safe attenuated strain of vaccinia, known as modified vaccinia virus, Ankura (MVA) was developed [8,9], but it was only used as primary vaccination followed by traditional smallpox vaccination. Moreover, its efficacy against smallpox was never directly tested due to the eradication of smallpox shortly thereafter and the question remains as to whether it would induce full or only partial immunity when faced against fully virulent smallpox. Although the virology, pathology, and epidemiology of smallpox are well described [1], there is a relative dearth of information regarding the immunology of smallpox and smallpox vaccination. Most importantly, there is currently no consensus on the immunological correlates of protection, making it difficult to implement rationale vaccine design when it not established which immunological benchmarks are necessary for full or even partial protection. Recent quantitative analysis of the cellular and humoral immune response following smallpox vaccination, coupled with historical evidence of protective immunity, are beginning to shed light on this important subject.

## Discussion

### The question of safety following smallpox vaccination

The current smallpox vaccine was prepared prior to 1982 under standards that today, would be unlikely to be approved by the FDA. To produce the vaccine, the torso of bovine calves were shaved, and their skin scarified (scratched) with an inoculum containing vaccinia virus. After the infection has reached a point in which a great deal of exudate was observed, the purulent lymph material was scraped from the infected cow, clarified, and lyophilized in the presence of antibiotics. The inoculum is later reconstituted with diluent containing 0.25% phenol to further decrease bacterial contamination (<200 viable bacterial/mL after reconstitution; see Dryvax package insert). Unlike most other vaccines that are administered by subcutaneous or intramuscular injection, vaccinia virus replicates poorly under these conditions and optimal vaccination occurs by scarification of the virus inoculum onto the skin surface [10]. Viral replication on the skin surface typically results in a vesicular or pustular lesion that is described as a "take" which later crusts over and sloughs off, leaving behind a small scar. Since the time of Edward Jenner, the presence of a vesicular or pustular lesion has remained the gold-standard measurement of successful vaccination.

The success of smallpox vaccination does not come without unwanted consequences. The most common side effects of smallpox vaccination are fever and other flu-like symptoms. More serious adverse events include inadvertent inoculation (529 cases/10^6 ^doses), generalized vaccinia (242 cases/10^6 ^doses), eczema vaccinatum (39 cases/10^6 ^doses), vaccinia necrosum (1.5 cases/10^6 ^doses), encephalitis (12 cases/10^6 ^doses), or death (~1 death/10^6 ^doses) [11]. The mortality rate is highest for infants that are <1 year of age (5 deaths/10^6 ^doses) whereas the mortality rate for older children and adolescents aged 1–4 or 5–19 is approximately 0.5 deaths/10^6 ^doses (see also [12,13] and Dryvax package insert). It is rare for adults to die after smallpox vaccination; of 68 deaths attributed to smallpox vaccination over a 9-year period of evaluation, only 8/68 (12%) of cases occurred in adults. Five of the eight adults were over the age of 60 and 4/8 of the lethal cases occurred in adults who were diagnosed with terminal cancer at the time of vaccination [11].

Myopericarditis is a recently identified adverse event that occurs at a rate of ~124 cases/10^6 ^smallpox vaccinations [14,15]. In 2003, there were three fatal heart attacks that were temporally related to smallpox vaccination and this triggered critical evaluation of any vaccine-related cardiac events thereafter. It was later realized that all three heart attack victims (age 55, 55, and 57) had pre-existing risk factors for cardiac disease including hypertension, hyperlipidemia, and smoking. At autopsy, none of the three heart attack victims showed signs of myo/pericarditis but the deaths were instead linked directly to ischemic events [16]. A retrospective study analyzing the number of cardiac deaths among approximately 80,000 death certificates issued near the time of a massive smallpox vaccination effort in New York City in 1947 has also brought new insight into the dangers of smallpox vaccine-induced myocarditis[16]. Following an imported case of smallpox, about 6.4 million people of all ages were vaccinated in a one-month period. Analysis of the frequency of cardiac deaths before, during, and after the vaccination campaign failed to show a statistical increase in these events. Moreover, analysis of 64 recently identified cases of myocarditis in the U.S. military smallpox vaccination program found that approximately 80% of patients reported no long term sequelae and 100% of patients demonstrated objective normalization of echocardiography, electrocardiography, laboratory testing, graded exercise testing, and functional status [15].

To overcome some of the problems associated with the current calf lymph smallpox vaccine (Dryvax), an improved tissue culture-derived vaccine has been recently developed [17]. Unlike Dryvax, this new vaccine (designated ACAM1000) is produced under sterile GMP conditions, so bacterial contamination is avoided. Also, unlike Dryvax which contains a heterologous mixture of virus variants with some differing in their neurovirulence [17,18], ACAM1000 is clonally derived (triple plaque-purified) and tested extensively for low neurovirulence in animal studies. Thus, it is hopeful that ACAM1000 vaccine will have reduced risk of encephalitis, one of the major risk factors following smallpox vaccination. It remains to be seen whether or not myopericarditis or other potentially serious adverse events will be affected by the use of this new vaccine.

Both Dryvax and ACAM1000 represent replication-competent smallpox vaccines and since these vaccines are based on the use of live viruses, there is always an inherent risk of severe adverse events or death (albeit rare) in vaccinees that have unknown or undisclosed immunodeficiencies at the time of vaccination. To overcome the hazards of replicating viruses, a highly attenuated strain of vaccinia, designated Modified Vaccinia virus Ankara (MVA) was developed by growing the virus for >500 passages on chicken embryo fibroblasts (CEF) and following the loss of about 15% of its parental genome, it no longer was capable of replicating in most mammalian cells, including human cells [19]. Other strains of non-replicating orthopoxviruses have also been developed, including NYVAC, which was derived from vaccinia virus following a deletion of 18 genes – including those encoding virulence factors and human host range replication, and ALVAC, an attenuated viral vector that is based on canarypox, an avipoxvirus that grows only in avian species. In one study, recombinant NYVAC and recombinant ALVAC expressing JEV proteins were found to be well tolerated but more reactogenic than the commercially available formalin-inactivated JEV vaccine [20]. Of these attenuated poxvirus vaccine strains, MVA is the one with the most extensive history of safety in humans. Beginning in 1968, >100,000 people in Germany were vaccinated with MVA (followed by traditional smallpox vaccination) and although it was well tolerated, MVA was not used alone and since there were no smallpox outbreaks at that time, its efficacy in the face of an actual smallpox outbreak has not been tested. With an excellent safety profile in humans and in animal models of immunodeficiency, recombinant MVA expressing candidate immunogens from a variety of infectious agents (e.g. HIV, HPV, and malaria) or tumor antigens (e.g. melanoma) have now reached Phase I and Phase II clinical trials [21,22]. However, the role of MVA in the future of smallpox vaccination has yet to be decided and will likely be determined by the outcome of clinical trials that directly compare the immunogenicity of MVA to either Dryvax or ACAM1000, two vaccines that are likely to provide the required high levels of protective immunity that will be necessary in the event of an accidental or deliberate smallpox outbreak.

### Quantitative analysis of vaccine efficacy

Edward Jenner developed the first test for vaccine efficacy when he immunized subjects with cowpox and then later challenged them with smallpox by inoculation. If a subject showed no secondary smallpox lesions indicative of systemic spread, then the individual was believed to have protective immunity. He noted that cowpox-vaccinated individuals who developed a pox-like lesion at the vaccination site were fully protected against smallpox challenge. To this day, most studies still use the identification of a "take" (vesicular or pustular lesion) as evidence of successful vaccination. However, there are rare cases of revaccinated subjects who present with vesicle formation after smallpox vaccination, but show no detectably boosted cellular or humoral immunity [23] and M.K. Slifka, unpublished results). For this reason, it is important that pre- and post-vaccination serum antibody and peripheral T cell responses be monitored in individuals who plan to work with virulent orthopoxviruses or who would be expected to enter a hot zone in the case of a smallpox outbreak.

Quantitative immunology is beginning to gain acceptance as a measurement of vaccine efficacy, although the Jennerian vesicle at the site of smallpox vaccination still remains the primary endpoint of successful vaccination in most studies [17,23-25]. One of the reasons why quantitative immunology is more important now than ever before is that with some vaccines such as MVA, there is no vesicle formed due to the route of immunization – so unless immunological measurements are made, then vaccine immunogenicity cannot be determined or compared. Humoral immunity following smallpox vaccination was measured in the 1960's and 1970's by means of neutralizing assays (primarily against the IMV form of vaccinia or smallpox) and today, humoral immune responses are quantitated by analysis of neutralizing activity against IMV or EEV forms of vaccinia or by the use of ELISA assays using whole-virus lysate and/or individual vaccinia IMV or EEV proteins. Moreover, the vaccinia-specific memory B cell response has also been recently studied [26] providing the first direct quantitation of this memory cell subset. Quantitation of the antiviral T cell response mounted after smallpox vaccination was not an option during the smallpox era because the tools and technology were not available for analysis of cellular immunity. In contrast, today there are now several sophisticated techniques that can be used to monitor antiviral T cell responses directly *ex vivo *including vaccinia-specific IFNγ ELISPOT assays [17,23,26-28], intracellular cytokine staining analysis (ICCS) [29-33], or peptide/MHC Class I tetramer staining [28,30]. Each of these techniques has high sensitivity and high specificity and each has both advantages and disadvantages. For instance, the IFNγ ELISPOT assay provides a highly sensitive calculation of IFNγ-producing cells, but one drawback is that it only allows detection of one cytokine at a time and the phenotype of the IFNγ-producing subset (CD4, CD8 or possibly NK cells) must be determined by purifying each population independently prior to the assay. The advantage of ICCS is that antiviral T cells can be quantitated based on the production of more than one cytokine and the phenotype of the responding lymphocyte subset is directly determined by flow cytometry. The main disadvantage of ICCS is that a relatively large number of cells are required in order to detect rare populations of virus-specific T cells. The advantage of using peptide/MHC tetramers is that CD8^+ ^T cells can be quantitated regardless of their cytokine profiles, but the main disadvantages with this approach include the lack of identified CD4^+ ^T cell/MHC Class II epitopes, the requirement for knowledge of the MHC haplotype of the subject, and the inability to measure the total antiviral T cell response, which may be directed against any number of immunodominant and subdominant peptide epitopes.

Direct quantitation of the antiviral immune response induced by smallpox vaccination has been critical for several recent advances in orthopoxvirus immunobiology – especially since smallpox has been eradicated and other human orthopoxvirus outbreaks are too small and sporadic (e.g. cowpox [34,35] or monkeypox [36,37]) to be feasible for field studies of protective efficacy. For example, Weltzin *et al*. [17] not only compared the neutralizing titers induced by Dryvax vs. ACAM1000 vaccines, but also compared vaccinia-specific T cell responses by IFNγ ELISPOT as well as by cytolytic T cell assays and proliferation assays that, although less quantitative than the ELISPOT assay, are nevertheless important for analysis of antiviral T cell functions. These techniques allowed the investigators to demonstrate non-inferiority of the new tissue culture-derived smallpox vaccine compared to the Dryvax vaccine that is currently in use.

Another study by Earl *et al. *[38], used quantitative immunology to compare vaccine efficacy of Dryvax, MVA followed by a Dryvax booster, and MVA followed by an MVA booster in a cynomolgus monkey (*Macaca fascicularis*) model of monkeypox infection. Using ELISA assays and neutralizing assays to measure vaccinia-specific antibody responses, and ICCS to measure antiviral T cell responses, the authors showed that antiviral immunity appeared similar between these three groups. Likewise, each of these groups were protected against lethal monkeypox challenge, although primates that only received MVA plus an MVA booster showed partial protection with 6/6 animals presenting with 1–36 monkeypox lesions (compared to >500 monkeypox lesions in the unvaccinated controls). This indicates that two MVA vaccinations are required to induce partial immunity whereas MVA followed by Dryvax immunization or a single Dryvax immunization each provides full immunity. The protective efficacy of a single dose of MVA is unknown, but based on the results of the Earl *et al. *study [38] wherein two doses were required to elicit partial immunity, it appears likely that a single dose of MVA would be of only low protective value in the face of a virulent orthopoxvirus infection. This is not surprising since MVA, NYVAC, and ALVAC (all replication-deficient vaccines), typically require booster doses to be administered in order to elicit optimal immune responses. Moreover, several prime-boost strategies (including DNA vaccination followed by MVA booster) are being tested in order to overcome the low immunogenicity of MVA alone [21]. This is different from most replicating viruses [39] including vaccinia, which require only one immunization (or infection) to induce optimal and often lifelong immunity [5,26,32,40]. Approximately half of the U.S. population has been vaccinated against smallpox and continue to maintain pre-existing antiviral immunity [26,32] and this may have an impact on the efficacy of MVA vaccination. For instance, studies involving the closely related, non-replicating NYVAC strain have found that pre-existing immunity significantly effected the outcome of vaccination [20]. In this particular study, immunization with recombinant NYVAC expressing JEV proteins failed to induce protective antibodies in 0/5 vaccinia pre-immune individuals and although 5/5 vaccinia-naive subjects seroconverted after NYVAC-JEV immunization, the resulting neutralizing titers were substantially lower than that observed in subjects who received the standard formalin-inactivated JEV vaccine [20]. Oddly, the authors noted that both the NYVAC-JEV and the ALVAC-JEV vaccines (2 doses administered 28 days apart) failed to induce a detectable anti-vaccinia neutralizing response.

It is difficult to speculate the efficacy of MVA vaccination of humans as a method of protection against smallpox. It is possible that booster doses of MVA would provide strong enough immunity to at least protect against lethal smallpox, similar to its ability to protect primates from lethal monkeypox [38]. On the other hand, clinical trials could indicate that MVA vaccination alone may be too inconsistent or only induce low levels of immunity that would be considered inferior to live smallpox vaccination with calf-lymph or tissue culture-derived vaccine preparations. Unlike live smallpox vaccination, which can be used as an effective post-exposure treatment against the lethal consequences of smallpox [1,5], it is unlikely that the low immunogenicity of MVA would be capable of fulfilling this role. Moreover, in the event of a smallpox outbreak there would not be enough time to administer two or more doses of MVA if people were at a high risk of exposure. Under these circumstances, use of live viral vaccines would be critical for ring vaccination or mass vaccination scenarios. This is not to say that MVA would not have a potential role in biodefense strategies. For instance, in a pre-event scenario, one could foresee the use of MVA followed by vaccination with a live viral vaccine such as Dryvax or its equivalent. Under these circumstances, MVA would likely induce partial immunity that would reduce the adverse events that are associated with traditional smallpox vaccination. Moreover, MVA is the vaccine of choice in immunocompromised individuals with suppressed immune systems (cancer patients, organ-transplant patients, AIDS patients, etc.) who would otherwise be contra-indicated for administration of the live viral vaccines. More studies will be needed to determine the immunogenicity and protective efficacy of MVA and other related non-replicating vaccines in terms of their potential to counter a smallpox outbreak.

### The need for formal definition of protective immunity and the correlates of immunity

There is more than one definition of protective immunity against smallpox. There is protection against infection, protection against disease, and protection against death. Of these, one might argue that protection against lethal infection is the ultimate definition of protective immunity. However, protection against infection and protection against disease not only reduce the morbidity of an outbreak, but these high levels of protective immunity are also associated with reduced virus spread to others (Chapter 4, pages 189–90 of [1]). Protection against infection is the most rare level of protective immunity since this requires that an infection be blocked at the point of entry. A meta-analysis of 10 epidemiological studies on smallpox noted that on average, the virus only infected ~4% of previously vaccinated household contacts (Chapter 4, pages 189–90 of [1]). However, these results were based on whether or not the vaccinated contacts showed disease symptoms and did not necessarily prove that they were never infected *per se*. Further analysis indicated that approximately 10% of previously vaccinated household contacts of smallpox patients were actually infected with smallpox as demonstrated by isolation of infectious virus from pharyngeal mucosa, but only 4/34 (12%) of these subjects developed the clinical symptoms of smallpox [41]. Moreover, another study showed that about 50% of previously vaccinated, disease-free contacts demonstrated serological results indicative of a recent orthopoxvirus infection [42]. This suggests that most instances of "protection against infection" may not be complete protection. Instead, many of the individuals thought to have had protection against infection may have actually been infected with smallpox but didn't know it because they were clinically asymptomatic. Similar to these historical studies, during the U.S. monkeypox outbreak in 2003 [37] we have identified three previously unreported cases of monkeypox in subjects who had received smallpox vaccination many years earlier and were unaware that they had become infected with monkeypox because they were spared any recognizable disease symptoms (M.K. Slifka, unpublished data).

A major issue in the smallpox field is that there is no consensus on what is exactly required for protective immunity against this disease. In the age of quantitative immunology, we are beginning to find clues that might help answer this age-old question. In a study of >300 subjects, the levels of vaccinia-specific serum antibody and antiviral T cell responses were determined from 30 days to up to 75 years after smallpox vaccination [32]. Antiviral antibody responses were maintained essentially for life, whereas antiviral CD4^+ ^and CD8^+ ^T cell responses declined with a half-life of approximately 8–15 years, with CD4^+ ^T cell memory being more stable than CD8^+ ^T cell responses. Similar duration of antibody production was demonstrated by other recent studies as well as older literature [24,26,40]. Moreover, the gradual loss of T cell memory was also confirmed [26], as was the differential loss of CD8^+ ^T cell memory over CD4^+ ^T cell memory [33]. Based on historical analysis of vaccine-mediated protection against lethal smallpox (dating back to the age of Edward Jenner), indicates that protective immunity is often lifelong [40]. One might argue that if protective immunity against smallpox had an absolute requirement for antiviral CD4^+ ^or CD8^+ ^T cells, then protective immunity would not be life-long but would instead be more likely to decline at the same rates as T cell memory. This suggests that in humans, humoral immunity might play a more important role in protection against lethal infection than cellular immunity [5,40]. Smallpox disease symptoms become more pronounced with increased time since vaccination [43], and it likely that the combination of intact cellular and humoral immunity together provide the most robust antiviral immunity. Recent vaccinia studies in mice using either antibodies to deplete T cell subsets or mouse strains that are genetically deficient in CD4^+ ^T cells or CD8^+ ^T cells (or both) have indicated that, as long as there is strong humoral immunity against vaccinia, T cell memory is dispensable for protective immunity [44-46]. This result corresponds well with other studies in which protection against vaccinia or smallpox has been clearly demonstrated by the adoptive transfer of immune serum in humans or by transfer of monoclonal neutralizing antibodies in animal models [5,40]. On the other hand, adoptive transfer of virus-specific T cells and experiments in mice that are genetically deficient in B cells, also indicate that in the absence of pre-existing antibody responses, memory T cells can play an important role in protection. This indicates that cellular and humoral immunity have overlapping roles in protective immunity and one may compensate for a deficiency in the other.

Based on the overlapping roles for T cell and B cell memory on protective immunity, is there any chance that a consensus can be reached in regard to defining an immunological correlate of immunity? One way to address this issue is to examine historical studies in which an immunological correlate was identified. In this regard, there are two independent studies in which investigators showed that subjects with vaccinia-specific neutralizing antibody titers of >1:20 [47] or ≥ 1:32 [48] were fully protected against smallpox. The latter study was the largest of the two and showed that 3/15 (20%) of subjects with titers below 1:32 contracted smallpox whereas 0/127 (<1%) of subjects with antibody titers of ≥ 1:32 contracted the disease. The only caveat to these studies is that the subjects also received post-exposure vaccination at the same time that serum samples were drawn, so the full protection afforded to the subjects with high pre-existing neutralizing titers may have been due to their high antibody titers, or the combination of strong pre-existing antibody titers in the context of post-exposure vaccination. A neutralizing titer of 1:32 is equivalent to a vaccinia ELISA titer of 944 Elisa Units (EU) or approximately 4 International Units (IU) of the WHO/NIBSC International Smallpox Serum Standard [32]. Interestingly, about 50% of subjects vaccinated in the distant past maintain neutralizing titers of 1:32 or greater for life and this coincidentally is the same proportion of vaccinated smallpox contacts who demonstrated fully protective immunity when exposed to smallpox-infected family members [42]. This leads one to speculate that 4 IU may constitute a protective level of serological immunity against smallpox. This is a testable hypothesis and to determine if this is a correlate of protection, it will be important to perform adoptive transfer of VIG or its equivalent into non-human primates (resulting in antiviral serum antibody levels of approximately 1:32) and determine if they are protected against a lethal orthopoxvirus infection, such as following monkeypox challenge. This experiment would prove or disprove the hypothesis that a serological correlate of protective immunity exists and may lay the foundation for future vaccine design. Of note, the protective level of yellow fever immunity (log_10 _≥ 0.7 neutralizing titer) was also established in non-human primates by simply vaccinating groups of animals with different doses of the yellow fever vaccine, quantitating vaccine-induced antibody levels, and then challenging them with a lethal dose of yellow fever virus [49]. This serological correlate of protection (which ignores the role of vaccine-induced T cell responses) has been established as the benchmark of protective yellow fever virus-specific immunity for over 30 years and demonstrates the potential for using animal models to correlate protective immunity in humans.

## Conclusion

Traditional smallpox vaccination has lead to the global eradication of smallpox but continues to be used today in an effort to thwart the potential use of smallpox as a biological weapon. Since this vaccine employs the use of a live virus, there is an inherent risk of adverse events, although these are generally quite rare. New generation smallpox vaccine candidates include MVA and other non-replicating poxviruses and although they demonstrate a high degree of safety, their immunogenicity appears to be substantially lower than traditional smallpox vaccination with live vaccinia virus. The role of MVA and traditional smallpox vaccination (or a combination thereof) in future vaccination campaigns has yet to be determined. However, developing a consensus on the definition of what is required for protective immunity and defining an immunological correlate of immunity would aid in the evaluation of current and future vaccine approaches.

## List of Abbreviations

MVA modified vaccinia virus Ankura

GMP good manufacturing practices

HIV human immunodeficiency virus

CEF chicken embryo fibroblasts

HPV human papilloma virus

FDA Federal Drug Administration

JEV Japanese Encephalitis Virus

IMV Intracellular mature virus

EEV Extracellular enveloped virus

ELISA Enzyme-linked immunosorbent assay

IFNγ Interferon-gamma

ELISPOT Enzyme-linked immunosorbent Spot assay

ICCS Intracellular cytokine staining

MHC Major histocompatibility complex

VIG Vaccinia immune globulin

WHO World Health Organization

NIBSC National Institute of Biological Standards and Control

EU Elisa Units

IU International Units

## Competing Interests

OHSU and Dr. Slifka have a financial interest in Najít Technologies, Inc., a company that may have a commercial interest in the results of this research and technology. This potential conflict was disclosed to the OHSU Conflict of Interest in Research Committee and an approved management plan was implemented.
